# Customized Barrier Membrane (Titanium Alloy, Poly Ether-Ether Ketone and Unsintered Hydroxyapatite/Poly-l-Lactide) for Guided Bone Regeneration

**DOI:** 10.3389/fbioe.2022.916967

**Published:** 2022-06-28

**Authors:** Yilin Shi, Jin Liu, Mi Du, Shengben Zhang, Yue Liu, Hu Yang, Ruiwen Shi, Yuanyuan Guo, Feng Song, Yajun Zhao, Jing Lan

**Affiliations:** ^1^ Department of Implantology, School and Hospital of Stomatology, Cheeloo College of Medicine, Shandong University, Jinan, China; ^2^ Shandong Key Laboratory of Oral Tissue Regeneration, Jinan, China; ^3^ Shandong Engineering Laboratory for Dental Materials and Oral Tissue Regeneration, Jinan, China

**Keywords:** guided bone regeneration, customized, barrier membrane, titanium alloy, polyether ether ketone, unsintered hydroxyapatite/poly-l-lactide

## Abstract

Sufficient bone volume is indispensable to achieve functional and aesthetic results in the fields of oral oncology, trauma, and implantology. Currently, guided bone regeneration (GBR) is widely used in reconstructing the alveolar ridge and repairing bone defects owing to its low technical sensitivity and considerable osteogenic effect. However, traditional barrier membranes such as collagen membranes or commercial titanium mesh cannot meet clinical requirements, such as lack of space-preserving ability, or may lead to more complications. With the development of digitalization and three-dimensional printing technology, the above problems can be addressed by employing customized barrier membranes to achieve space maintenance, precise predictability of bone graft, and optimization of patient-specific strategies. The article reviews the processes and advantages of three-dimensional computer-assisted surgery with GBR in maxillofacial reconstruction and alveolar bone augmentation; the properties of materials used in fabricating customized bone regeneration sheets; the promising bone regeneration potency of customized barrier membranes in clinical applications; and up-to-date achievements. This review aims to present a reference on the clinical aspects and future applications of customized barrier membranes.

## 1 Introduction

Sufficient bone volume is indispensable to achieve functional and aesthetic results in the fields of oral oncology, trauma, and implantology (Matsuo et al., 2010). The functional reconstruction of jawbone defects remains a major clinical challenge (Major et al., 2020; Kondo et al., 2022), and thus the effective repair and regeneration of maxillofacial and alveolar bone has great significance for maxillofacial reconstruction and oral function. Rapid developments in biomedical materials science and the continuous innovation and improvement of surgical procedures in clinical practice have increased the predictability of maxillofacial and alveolar bone reconstruction, and treatment options have increased. Currently, a variety of materials and surgical techniques have been applied to vertical and horizontal bone augmentation, including bone-grafting techniques (Yamada and Egusa, 2018; Mangano et al., 2021), guided bone regeneration (Amaral Valladão et al., 2020) and distraction osteogenesis (Dimitriou et al., 2011). Each method varies and should be implemented according to clinicians’ experiences and specific situations.

In guided bone regeneration (GBR), one of the most predictable methods for reconstructing maxillofacial and alveolar bones, a membrane is used to isolate soft tissues and thereby promote bone regeneration. The application of barrier membranes is a key factor in the success of GBR (Angelo et al., 2015). To date, a variety of barrier membranes have been developed to perform multiple functions in clinical applications and can be divided into resorbable or non-resorbable membranes (Rakhmatia et al., 2013). Resorbable barrier membranes, such as collagen membranes, are widely used clinically because they have high biocompatibility and no need for a second surgery to be removed (Lee et al., 2022). However, uncontrolled degradation, insufficient stiffness, and space maintenance often lead to inadequate bone regeneration (Zhou et al., 2021). Non-resorbable barrier membranes, such as titanium (Ti) mesh, have excellent mechanical properties, which can provide space for bone regeneration and reduce the volume of bone grafts. However, a traditional Ti mesh does not conform to the anatomical shape of a bone defect area, and the intraoperative cutting and bending of the Ti mesh may increase the risk of postoperative exposure and repeated mucosal irritation (Jung et al., 2014). Therefore, customized, three-dimensional (3D), and preformed barrier membranes with favorable mechanical properties are needed for ideal bone regeneration. Advances in modern 3D computer-aided planning and the application of computer-aided design or computer-aided manufacturing (Oberoi et al., 2018) have facilitated the fabrication of customized titanium (Ikawa et al., 2016), poly ether-ether ketone (PEEK) (El Morsy et al., 2020), and unsintered hydroxyapatite/poly-l-lactide (uHA/PLLA) (Matsuo et al., 2010) meshes to closely fit the anatomical shapes of bone defect areas for the accurate reconstruction of the 3D volume and position of the jaw (Vaquette et al., 2021).

**FIGURE 1:**
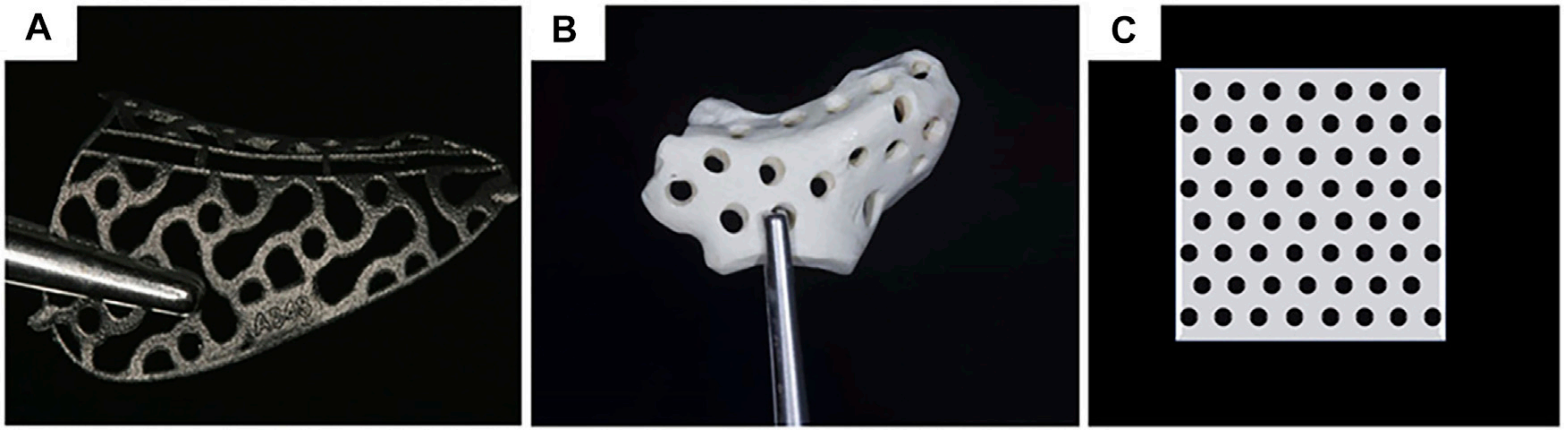
Materials for customized mesh: **(A)** titanium alloy (Hartmann and Seiler, 2020); **(B)** PEEK (Mounir et al., 2019); **(C)** uHA/PLLA. Reproduced with permission from (Hartmann and Seiler, 2020) and (Mounir et al., 2019).

This article outlines the basic workflow and advantages of modern 3D computer-aided surgery and critically analyzes materials (titanium alloy, PEEK, and uHA/PLLA; Figure 1) in the fabrication of customized barrier membranes, focusing on their uses in maxillofacial reconstruction and alveolar bone augmentation. Current developments in biomedical materials science and clinical aspects and future applications of patient-customized barrier membranes are discussed.

## 2 Protocols and Advantages of Modern 3D Computer-Assisted Surgery

**FIGURE 2:**
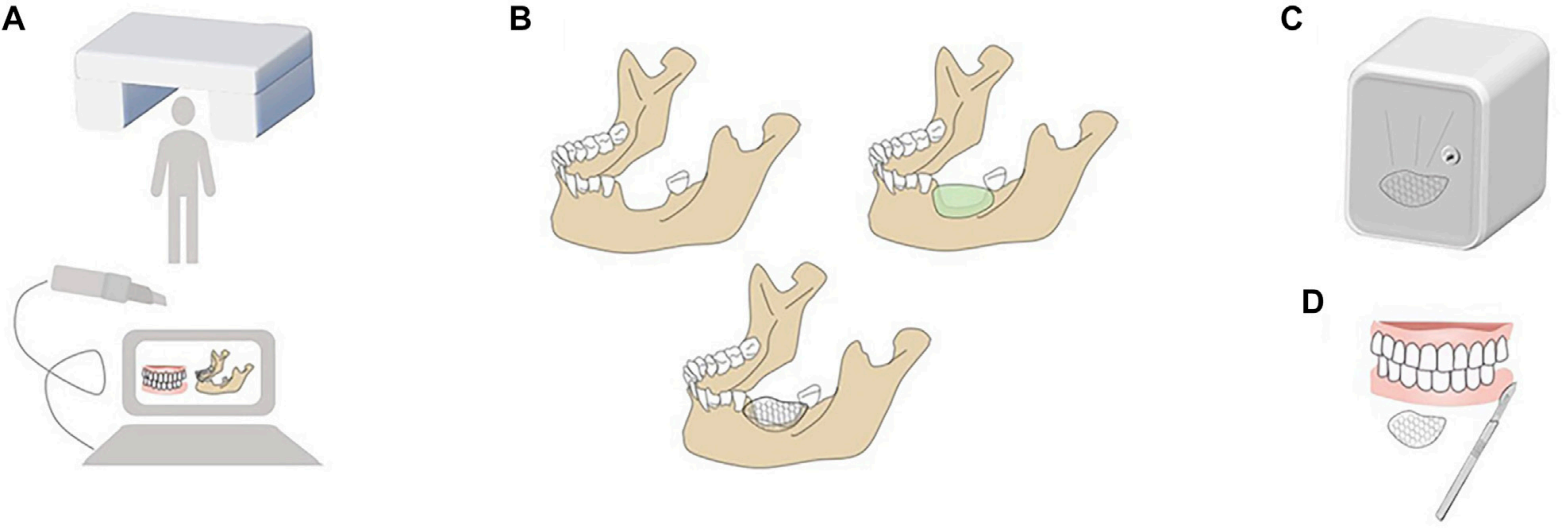
Protocols of modern 3D CAS. **(A)** Information acquisition: Information is required by intraoral scanning and digital imaging and communications in medicine (DICOM) recording remaining alveolar bone, positioning of critical anatomic structure and soft tissue condition. **(B)** Planning and Virtual operation: Planning is to design the optimal scheme according to the size and range of defects virtually; Virtual operation is to define the best osteotomy boundary or bone grafting range according to the tumor boundary or defect condition in the software to realize virtual positioning design. **(C)** 3-D printing: 3D printing is an additional manufacturing technique that deposits materials layer by layer to construct predesigned models. **(D)** Surgery and Postoperative Analysis: Precise osteomy and accurate localization of the implant are prerequisites of successful operation. And postoperative CT scan is necessary to evaluate the designed and actual results and analyze why deviations occur and how to deal with them.

Modern 3D computer-assisted surgery (3D CAS), which combines 3D printing technology with 3D imaging techniques, has undergone remarkable developments in the past decades. As shown in Figure 2, several protocols are essential to 3D CAS: 1) information acquisition, 2) planning, 3) virtual operation, 4) 3D printing, and 5) surgery and postoperative analysis (Troulis et al., 2002; Prevost et al., 2019; Mian et al., 2022). Although potential errors occur in the fabrication of customized barrier membranes after tomography data acquisition, image processing, and 3D fabrication (Tian et al., 2021), technological developments in rapid prototyping systems have considerably contributed to the accurate and detailed replication of craniofacial devices (Sharma et al., 2021). A previous study scanned skulls using tomographic imaging and prototyped them through selective laser sintering and using a 3D printing technology. Comparison with the original skulls and analysis showed an error of only 2.10% for selective laser sintering and 2.67% for 3D printing. Stoop et al. obtained cone beam computerized tomography (CBCT) images of six patients with alveolar bone deficiencies. After prototyping their alveolar bone models and 3D printing customized resin grafts, they evaluated the fitness of the resin grafts to alveolar bone models and found that the mean marginal fit of the resin grafts was better in small defect zones (0.46 ± 0.20 mm) than in large defect zones (0.52 ± 0.18 mm) and all met clinical requirements (Silva et al., 2008, Stoop et al., 2019). The above results showed that data analysis and processing using CBCT allows the visualization of maxillofacial structures (Vannier et al., 1984) and facilitates analysis of bone abnormalities. 3D printing technology can constitute customized barrier membranes that conform to complex tissue morphology (Kang et al., 2016). An efficient 3D CAS technology combining high-precision 3D printing and 3D imaging is a good option for alveolar bone augmentation and maxillofacial bone reconstruction.

**FIGURE 3:**
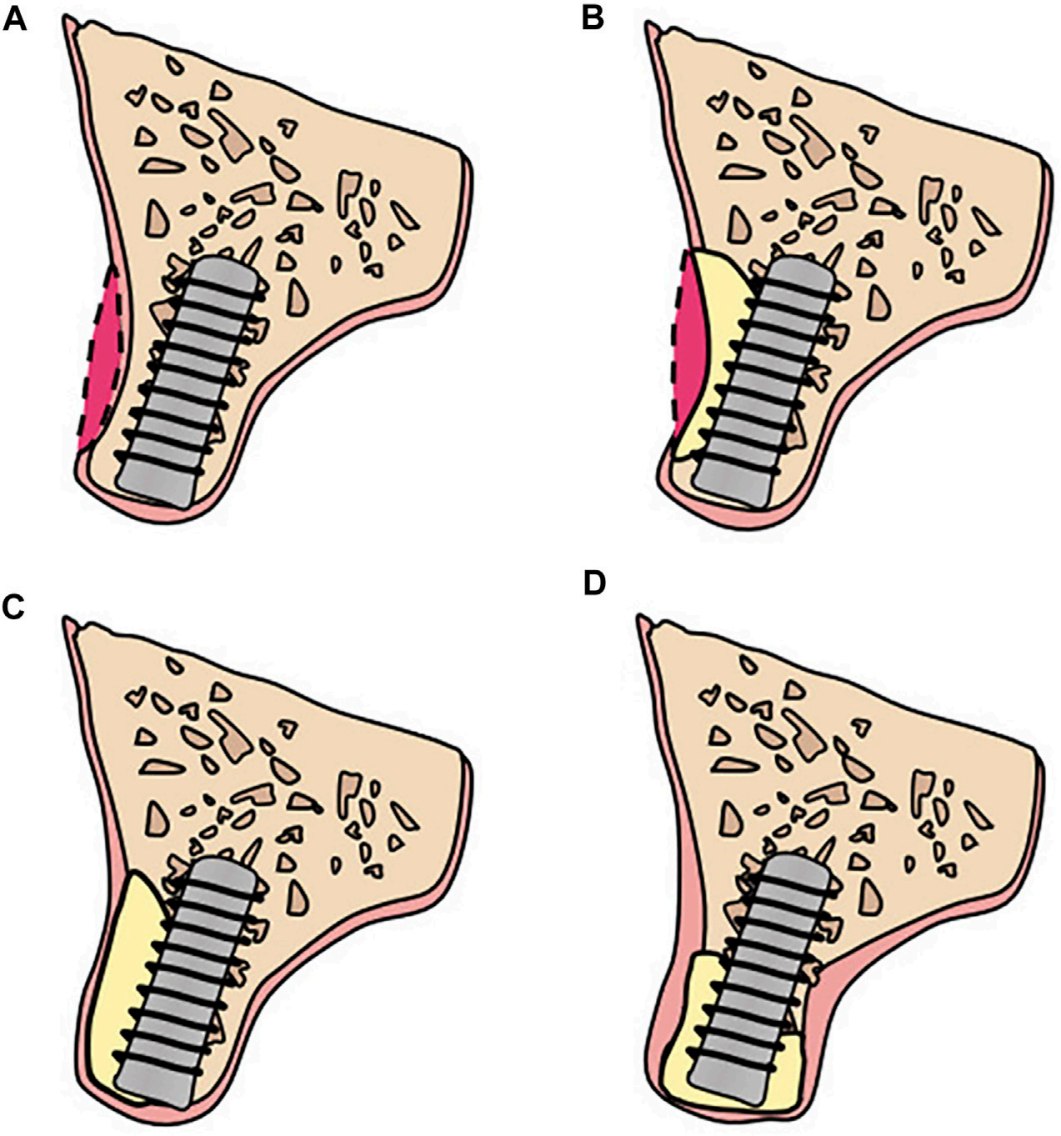
Bone defects classification: **(A)** Class 1: ideal alveolar bone condition: implants can be placed in an ideal, restoration-driven location without augmenting the volume of alveolar ridge. Although soft tissue grafts are sometimes recommended; **(B)** Class 2: a moderate horizontal atrophy: a dehiscence or a fenestration of the buccal plate is present. Implants are placed combined with hard tissue augmentation procedures; **(C)** Class 3: large degree of horizontal defects: residual alveolar ridge allows for a two-stage implant placement. Sufficient bone graft volume and adequate healing time are indispensable; **(D)** Class 4: severe atrophy on height and width: the remaining alveolar bone is in poor condition and there are commonly two alternatives: (i) onlay bone grafting; (ii) GBR with autogenous particulate bone and/or xenogeneic bone using Ti mesh.

According to the principle of “prosthetic guided regeneration,” alveolar bone defects can be divided into four classes, as shown in Figure 3 (Chiapasco and Casentini, 2018). For severe bone deficiency, Lizio et al. reconstructed 19 complex alveolar bone defects with a customized Ti mesh (Lizio et al., 2022). Morsy et al. rehabilitated 14 patients with severely atrophied alveolar ridges by using a customized PEEK mesh (El Morsy et al., 2020). Matsuo et al. applied customized uHA/PLLA meshes to patients with partial mandibular resection and achieved successful mandibular reconstruction (Matsuo et al., 2010). These successful cases indicate that customized barrier membranes in 3D CAS apply to a class 4 alveolar bone defect when a large horizontal or vertical bone defect or both occurs in an alveolar bone.

## 3 Optimization of Barrier Membranes

Barrier membranes in GBR act as protective films that maintain a stable bone regeneration environment, and an “ideal” barrier membrane is supposed to meet five basic principles (Scantlebury, 1993): biocompatibility (refers to the compatibility between hosts and biomaterials); space preservation (sufficient stiffness to support tissues above, maintain space, and withstand the pressure of mastication forces); selective permeability (prevention of soft tissue invasion while allowing osteogenic cells to proliferate); host tissue integration (for the embedding of surrounding host tissues); and clinical manipulation (ease of use and handling during clinical application). Through 3D printing and additional processes, the characteristics and biological performance of barrier membranes can be optimized by adjusting their microstructures given that their thickness, pore size, and roughness affect their mechanical performance and are directly related to bone formation ability.

### 3.1 Thickness

A customized membrane should have sufficient stiffness to create and maintain a suitable space for the intended bone regeneration, which is mainly related to the thickness of the membrane (Papia et al., 2022). A strong and thick membrane can withstand the pressures exerted by external forces, such as masticatory pressure. However, this also increases the risk of mucosal irritation and exposure (Levine et al., 2022). Therefore, determining the optimal thickness is crucial for customized membranes.

The thickness of Ti mesh commonly used ranges from 0.1 to 0.6 mm currently (Xie et al., 2020). For 3D printed Ti mesh, 0.4 mm-thick mesh is recommended, which can withstand sufficient strength and reduce irritation to mucosa, while 0.3 mm-thick mesh is suitable for the aesthetic zone with a missing tooth (Bai et al., 2019). PEEK, which has better tensile strength and elasticity, is closer to human bones than Ti and is widely used in cranioplasty (Panayotov et al., 2016). A previous study showed that a 0.6 mm-thick PEEK lattice with 2 mm pores could maintain space under masticatory pressure in the edentulous region (Li et al., 2022). For bioresorbable membranes, a certain thickness is essential for sufficient mechanical strength (Shikinami and Okuno, 1999). Studies have shown that a 0.8 mm-thick uHA-PLLA membrane could improve fine bone quality in mandibular reconstructions (Matsuo et al., 2010), and a single-folded 0.5 mm-thick uHA-PLLA membrane in orbital floor and medial wall reconstruction could facilitate the recovery of ophthalmologic function without causing intraoperative or post-operative complications (Kanno et al., 2017). However, there is no consensus on the thickness of the uHA-PLLA membrane for GBR. These results showed that thickness affects the stiffness of a barrier membrane. Optimal thickness is crucial to meeting malleability requirements while maintaining strength. Studies on the exposure rates of the three customized barrier membranes with different thicknesses are still lacking, and further studies are needed to make a compromise between thickness and membrane exposure.

### 3.2 Pore Size

The pores of barrier membranes are channels for nutrient and oxygen exchanges and are closely related to the blocking ability of cells. Nonporous barrier membranes can prevent fibroblasts from growing into bone defects, but insufficient blood supply may delay bone regeneration (Celletti et al., 1994; Guo et al., 2020). A pore size of >100 µm is a prerequisite for the penetration of blood vessel-rich tissues and soft tissue healing (Chvapil et al., 1969). A previous report based on three-point bending tests and finite element analysis suggested that a customized Ti mesh with a large diameter (3–5 mm) possessed suitable mechanical properties, while animal and clinical evidence of its osteogenic effect was lacking (Bai et al., 2019). Similarly, Li et al. reported that a 0.6 mm-thick PEEK lattice with 2 mm pores could maintain space under masticatory pressure in the edentulous region, but they did not compare the biomechanical properties and osteogenic effect of PEEK mesh with different pore sizes (Li et al., 2022). The optimized pore size for uHA-PLLA barrier membranes is still unclear. A large pore size ensures efficient blood supply to a wound and is critical to integrating membranes into surrounding tissues and stabilization of bone grafts, but it may enable more connective tissues to grow in. Pseudo-periosteum, a layer of fibrous connective tissue with few blood vessels, is always observed between a Ti mesh and bone (Gutta et al., 2009). Whether large pore size contributes to bone formation remains controversial. A large aperture may accelerate early bone repair but has no effect on the final bone mass (Zellin and Linde, 1996; Zhang H. Y. et al., 2019). There are also studies proving that macroporous membranes are more effective in promoting bone regeneration than microporous membranes (Gutta et al., 2009). The results indicate that the optimal pore size of membranes can be determined using advanced tools such as three-point bending tests and finite element analysis (Guo et al., 2021) to test the mechanical strength of different materials. Animal and clinical evidence are required to adjust the proper pore size to minimize soft tissue ingrowth without compromising nutrient exchange.

### 3.3 Roughness

Surface properties, especially roughness, can be easily manipulated using post-production surface treatments, such as acid etching, sandblasting, and electropolishing, and directly affect the interactions of implant–cell interfaces, which play an important role in cell responses, including adhesion, adsorption, and differentiation (Elias et al., 2008). Smooth surfaces can slow down biological processes at interfaces, and high roughness promotes bacterial adhesion and increases the possibility of implant failure. Optimal micro- and nano-roughness has been shown to promote osteoblast proliferation and differentiation (Rosales-Leal et al., 2009). For customized laser melting Ti mesh, an Ra value of 0.5 is recommended to promote osteoblast adhesion (Silva et al., 2009). Piotr Prochor et al. showed that an Ra value of 0.3 μm is the optimal roughness of a glass-reinforced PEEK and ensures high human osteoblast activity (Prochor and Mierzejewska, 2019). Meanwhile, the suitable roughness of uHA-PLLA for GBR is still unclear. To deliver the best possible clinical outcome, more studies are needed to define the optimal surface properties of 3D printed barrier membranes.

These studies suggest that a basic barrier membrane should possess abilities including biocompatibility, space preservation, selective permeability, host tissue integration, and clinical manipulation ability. The mechanical properties and biological performance of customized barrier membranes can be enhanced by optimizing their thickness, pore size, and roughness. Basic research is still needed as current research has not yet achieved a considerable balance between intrinsic properties and clinical needs.

## 4 Advances in 3D Printed Barrier Membrane for Guided Bone Regeneration

### 4.1 Customized Ti Mesh

#### 4.1.1 Properties of Customized Ti Mesh

The customized Ti mesh has good mechanical and biological properties. The physical traits of a selective laser melting (SLM) Ti mesh should be tested by tensile test, mean elongation strength, proof stress, or micro Vickers hardness to ensure impact and fracture stability (Sumida et al., 2015). Its high rigidity can maintain space and stabilize grafts, and its elasticity prevents the compression of the mucosa. Although a customized Ti mesh is thick and difficult to trim during operation (Silva et al., 2008), it conforms to alveolar bone morphology after selective laser sintering and 3D printing and prevents over-adjustment during surgery (Sumida et al., 2015). Moreover, corrosion resistance and biocompatibility ensure its stability (Her et al., 2012). Warnke et al. demonstrated the biocompatibility of patient-specific SLM Ti mesh at the cellular level through scanning electron microscopy, cell vitality staining, and biocompatibility testing (Warnke et al., 2009). Gyu-Un Jung et al. obtained fairly good bone regeneration results under a preformed Ti barrier membrane (Jung et al., 2014), and their histological results showed that newly regenerated bone was perfectly incorporated into the remaining allograft (Dellavia et al., 2021). These results indicate that a customized Ti mesh engineered with digital modeling technology and 3D printing technology is highly biocompatible, has the characteristics of high strength, good shape, high precision, simplicity, and convenience, can fit closely to the alveolar bone anatomy, and reconstruct the jaw precisely in terms of 3D volume and position.

#### 4.1.2 Fabrication, Clinical Application, Complications of Customized Ti Mesh

##### Fabrication

Customized Ti devices are developed with two methods. One method is bending a commercial Ti mesh on a 3D printed augmented alveolar bone model (Li S. et al., 2021). EI Chaar et al. proposed a similar method (El Chaar et al., 2019). First, they prototyped a preoperative alveolar bone model and then used wax to raise the alveolar ridge contour before bending the Ti mesh. They achieved considerable results in clinical use in terms of bone gain (5.94–6.91 mm horizontally and 5.76–6.99 mm vertically). Another approach is to design a containment mesh directly on a virtually designed model and prototype it with SLM. Compared with a folded customized Ti mesh, a SLM Ti mesh is thicker and more difficult to trim during operation (Silva et al., 2008).

In the design process, the formation of a pseudo-periosteum and fixation scheme need to be considered. The pseudo-periosteum is a layer of connective tissue that can be observed above the newly formed bone (Dahlin et al., 1998), which may be relevant for bone graft protection, prevention of infection, and absorption. At present, we have not found reports on the formation of pseudo-periosteum beneath PEEK and uHA/PLLA-based customized barrier membranes. Formation of pseudo-periosteum has been reported beneath the Ti-reinforced polytetra-fluoroethylene membrane and Ti mesh membrane or Ti mesh plus resorbable membranes (Cucchi et al., 2019b; Giragosyan et al., 2022). The cause of pseudo-periosteum is not clear, but there may be multiple factors: 1) insufficient cell exclusion ability of the barrier membrane due to its pores (Xie et al., 2020; Choi et al., 2021), 2) the local biological immunological response (Giragosyan et al., 2022), 3) chronic bacterial infections (Croes et al., 2019), 4) the local hypoxic microenvironment (Zhuang et al., 2022), and 5) micromovement between the bone and the membrane (Wang and Boyapati, 2006). As formation of pseudo-periosteum is inevitable, a new augmentation section should be over-contoured to 1.5 mm to offset the volume of the pseudo-periosteum beneath a Ti mesh (Ciocca et al., 2013). Songhang Li et al. added an additional predetermined thickness of 0.5 mm buccally and 1.0 mm vertically in precision bone augmentation (Li S. et al., 2021). The fixation of a Ti mesh is supposed to stabilize blood clots and protect bone augmentation areas. However, Ciocca et al. used no screws to immobilize a customized Ti mesh and still achieved considerable bone augmentation following good fixation (Ciocca et al., 2011).

These results show that pseudo-periosteum formation beneath a Ti mesh is inevitable. Besides, pseudo-periosteum can prevent infection and needs to be considered to design an over-contoured bone volume, and more research is needed in that area to truly understand its nature and importance to the guided bone regeneration process. A customized Ti mesh can be designed to precisely conform to the shape of an alveolar bone and fully engage existing undercuts, so few screws are required to ensure secure fixation.

##### Clinical Application

**FIGURE 4:**
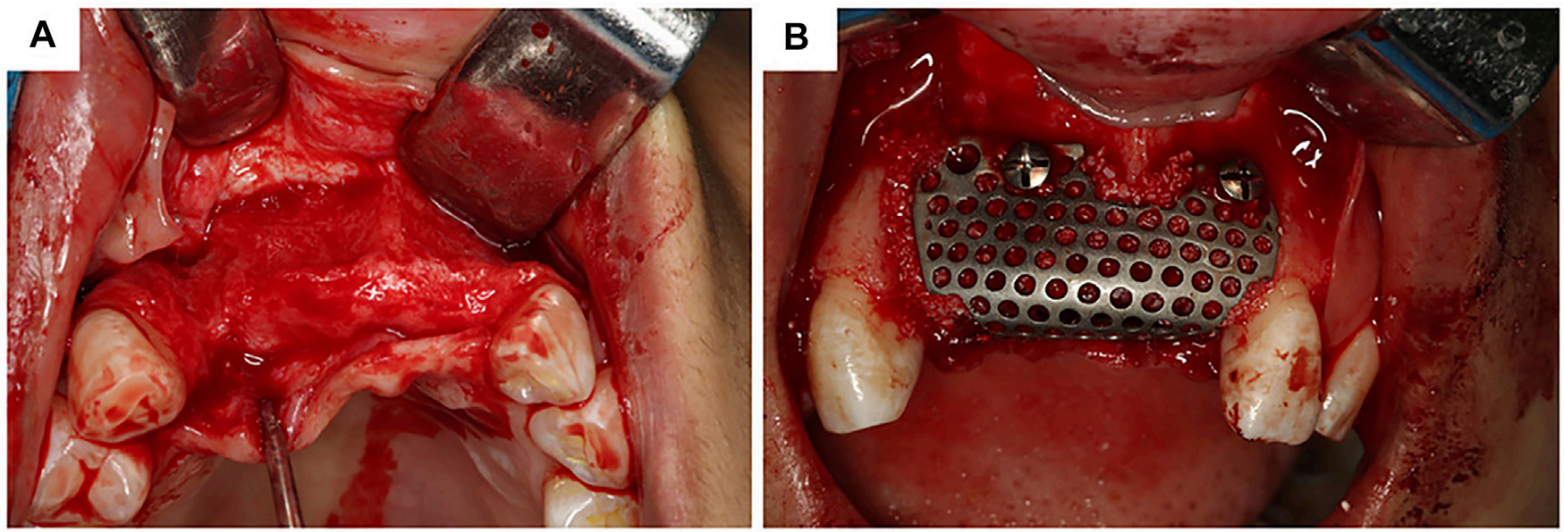
Customized titanium mesh in clinical usage. **(A)** Exposure of alveolar bone ridge. **(B)**Fixation of customized titanium mesh.

Ti and its alloys have been widely used in alveolar bone augmentation in atrophied posterior alveolar ridges and anterior aesthetic zones (Table 1; Figure 4). A study designed an L-shaped Ti mesh by preforming commercial Ti mesh on a model; a 2-year follow-up of 12 patients with a total of 16 implantation sites showed bone augmentation of 3.61 ± 1.50 mm vertically and 3.10 ± 2.06 mm horizontally (Zhang T. et al., 2019). Tallarico et al. have reported the successful application of customized Ti mesh in the anterior aesthetic area (Tallarico et al., 2020).

**TABLE 1:** Summary of clinical studies with customized mesh for GBR.

Category	Reference	Method for making mesh	No. of patients	No.of graft sites	Thickness	Pore size	Roughness	Cover materials	Complication	Bone augmentationoutcome
Titanium mesh	Ciocca et al. (2011)	Type 2	1	1	0.6 mm	Square 1.0 mm holes	NA	None	NA	AHB: 3.41 ± 0.89 mm; AVB: 2.57 ± 0.86 mm
	Sumida et al. (2015)	Type 2	13	13	0.3 mm	Round 1.0 mm hole	Mirror polished	None	Mucosal rupture: 7.7%	NA
	Sagheb et al. (2017)	Type 2	17	21	NA	NA	NA	6 cases with a resorbable collagen membrane	Mesh exposure: 33.3%	AHB: 5.50 ± 1.90 mm
								13 cases with a double layer of collagen membrane and platelet-rich fibrin membranes		AVB: 6.50 ± 1.70 mm
	Ciocca et al. (2018)	Type 2	9	9	0.1 mm	Round 1.0-mm hole	NA	None	Mesh exposure: 66.7%	MAB: 1.72–4.10 mm (mean: 3.83 mm)
										MB: 2.14–6.88 mm (mean: 3.95 mm)
	Al-Ardah et al. (2018)	Type 1	1	1	NA	NA	NA	Platelet-rich fibrin membranes	NA	NA
	Cucchi et al. (2019a)	Type 2	1	1	NA	NA	NA	Plasma rich in growth factors membranes	NA	NA
	El Chaar et al. (2019)	Type 1*	17	17	NA	NA	NA	None	Mesh exposure: 35.3%	AHB: 5.94 mm
									Mesh failure: 11.8%	AVB: 6.99 mm
	Ghanaati et al. (2019)	Type 2	7	NA	NA	NA	NA	Platelet-rich fibrin membranes	No signs of complications were observed in exposed open healing model	NA
	Takano et al. (2019)	Type 2	1	1	0.6 mm	NA	NA	None	NA	NA
	Hartmann et al. (2019)	Type 2	65	70	NA	NA	NA	Advanced- and injectable-platelet-rich fibrin and a collagen membrane	Mesh exposure: 37.1%	NA
	Hartmann and Seiler, (2020)	Type 2	55	68	NA	NA	NA	12 cases with advanced-platelet rich fibrin; 56 cases with a collagen membrane	Mesh exposure: 25.0%	Misch’s classification:D1 (17.6%), D2 (52.9%), D3 (19.1%) and D4 (10.3%)
	Cucchi et al. (2020)	Type 2	10	10	<0.5 mm	NA	NA	None	Mesh exposure: 10.0%	AVB: 4.5 ± 1.8 mm
	Tallarico et al. (2020)	Type 2	1	1	NA	NA	NA	None	NA	NA
	Hofferber et al. (2020)	Type 2	9	9	0.5 mm	NA	NA	Collagen membrane	Mesh exposure: 44.4%	AHB: 3.02 ± 0.84 mmAVB: 2.86 ± 1.09 mm
	Li et al. (2021b)	Type 1	21	22	NA	NA	NA	None	Mesh exposure: 9.1%	AHB: 4.11 mm (1.19–8.74)AVB: 2.48 mm (0.29–6.32)
	Li et al. (2021a)	Type 2	16	16	0.2 mm	Uniform apertures of 2.0 mm diameter	NA	Collagen membrane and concentrated growth factor matrix	Mesh exposure: 18.8%Wound dehiscence without mesh exposure: 6.3%	AHB:4.06 ± 2.37, 5.58 ± 2.65, and 5.26 ± 2.33 mm at levels of 0, 2, and 4 mm below the implant platform AVB: 3.55 ± 3.74 mm
	De Santis et al. (2021)	Type 2	5	12	NA	NA	NA	Collagen membrane	Mesh exposure: 8.3%	AHB: 3.60 ± 0.80 mmAVB: 5.20 ± 1.10 mm
	Chiapasco et al. (2021)	Type 2	41	53	NA	NA	NA	Collagen membrane	Mesh exposure: 20.8%	AHB: 6.35 ± 2.10 mmAVB: 4.78 ± 1.88 mm
	Nickenig et al. (2022)	Type 2	3	7	NA	NA	NA	Collagen membrane	No mesh exposure was observed	AHB: 3.70 mm (SD ± 0.59)
	Lizio et al. (2022)	Type 2	17	19	0.1–0.5 mm	NA	NA	None	Mesh exposure: 52.3%Mesh failure: 26.3%	Three-dimensional bone gain percentage: 88.2 ± 8.32% in 74% of the cases
PEEK mesh	Mounir et al. (2019)	Type 2	16	NA	2 mm	NA	NA	Collagen membrane	Mesh exposure: 1 case	Three-dimensional bone gain percentage:31.8 ± 22.7%
	El Morsy et al. (2020)	Type 2	14	NA	NA	NA	NA	None	Mesh exposure: 1 case	AHB: 3.42 ± 1.10 mmAVB: 3.47 ± 1.46 mm
uHA/PLLA mesh	Matsuo et al. (2010)	Type 1	2	2	0.8 mm	NA	NA	None	NA	Hounsfield unit value in new bone area was 790

Type 1, bend a commercial mesh on a 3D printed planned augmented alveolar bone model.

Type 1*, use wax to raise a preoperative alveolar bone model before bending the mesh.

Type 2, design the containment mesh directly on the virtually planned model and prototype it.

AVB, average vertical bone gain; AHB, average horizontal bone gain.

MAB, mandibular arch bone gain; MB, maxillary bone gain.

NA, not available.

In maxillofacial reconstruction, a customized 3D printed Ti mesh has been applied to patients undergoing total maxillectomy for the reconstruction of maxillary contour and rehabilitation of orbital floor and orbital volume and has produced satisfactory results with few complications, such as exophthalmos and diplopia (Liu et al., 2019). On the basis of the “Dumbach Titam Mesh-System” (Stryken-Leibinger, Freiburg, Germany; (Cheung et al., 1994), which first applied a commercially available Ti mesh tray and particulate cancellous bone marrow in mandibular discontinuity reconstructions and achieved considerable results, Won-bum Lee et al. reported a case of successful secondary mandibular reconstruction in a large bone defect after using a Ti mesh and particulate cancellous bone marrow after the failure of the fibula free flap technique; they obtained adequate bone augmentation for subsequent prothesis (Lee et al., 2018). The successful cases above demonstrate that a customized Ti mesh can reconstruct the jaw precisely in terms of 3D volume and position, offer a guarantee for the pre-operation plan, prevent manual shaping during operation, and greatly shorten operation time. It is suitable for various bone defects, especially complex large bone defects.

##### Complications

**FIGURE 5:**
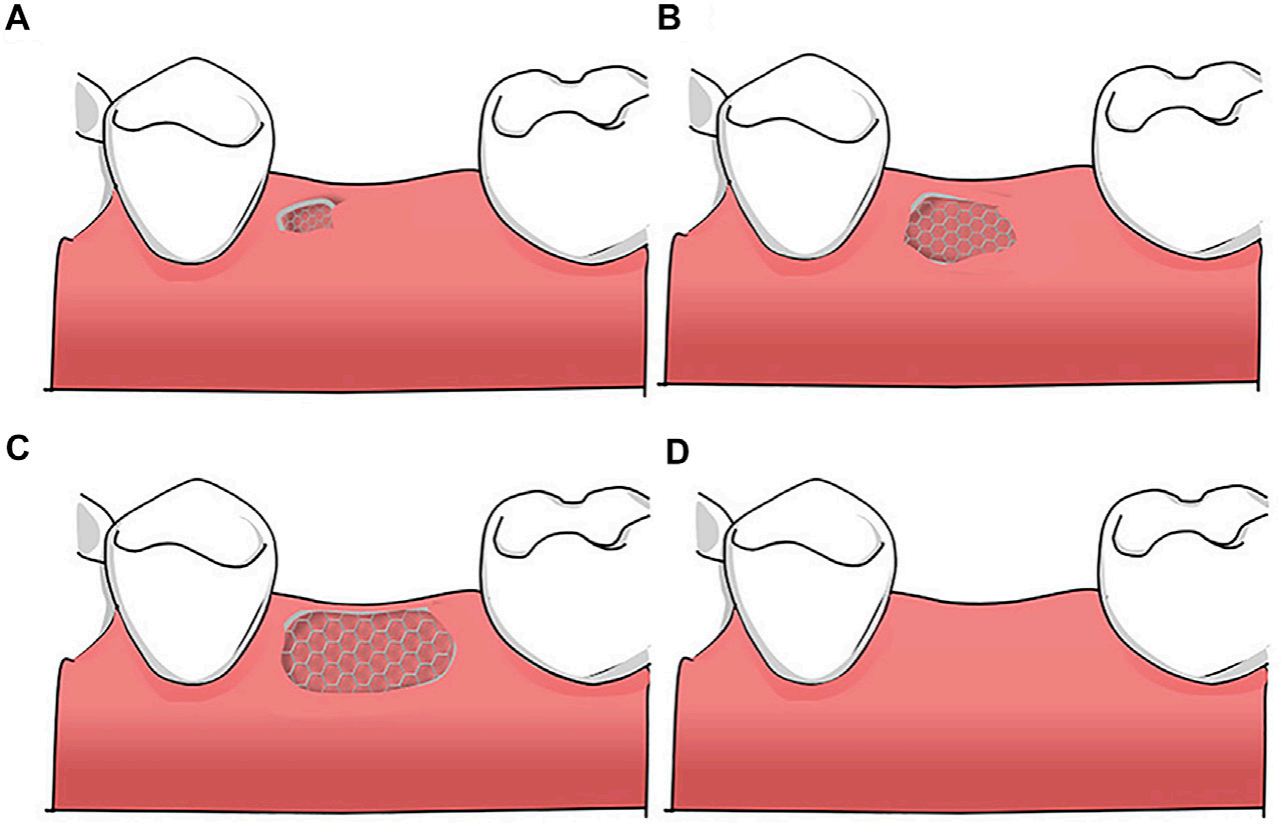
Classification of mesh exposure: **(A)** minor exposure; **(B)** one tooth width exposure (premolar); **(C)** exposure of an entire mesh **(D)** no exposure.

Ti mesh exposure is commonly associated with irritation to the mucosa because of its stiffness, sharp edges, and rough surfaces. Amely Hartmann et al. divided mesh and graft exposure into four classes (Hartmann et al., 2019): 1) minor exposure; 2) one tooth width exposure (premolar); 3) exposure of an entire mesh; and 4) no exposure (Figure 5). They found that age, periodontitis, diabetes, gender, tissue phenotype, and tobacco abuse are not associated with dehiscence probability, whereas tobacco abuse might accelerate graft loss when a mesh is exposed. These results are consistent with those of Lindfors et al., who showed that the success rate of bone augmentation was lower in smokers than in nonsmokers (Lindfors et al., 2010). A customized Ti mesh has a relatively low mesh exposure rate. In a clinical test, Sumida et al. equally divided 26 patients into two groups for alveolar bone augmentation surgery. One of the groups received customized Ti meshes, whereas the other received commercial Ti meshes. After post-operative follow-up, they observed that the exposure rates in the former (7.7%) were lower than those in the latter (23.1%). They attributed this result to the round and blunt shape of a customized Ti mesh (Sumida et al., 2015).

Dehiscence and mesh exposure can be prevented by properly managing soft tissues, and complete tension-free closure is indispensable during operation with single interrupted sutures and a deep mattress. Combination with collagen membranes does not reduce the exposure rate of a customized Ti mesh (Cucchi et al., 2021). Autologous bioactive materials, such as blood-based and platelet-rich plasma systems (Eppley et al., 2006; Torres et al., 2010), platelet-rich fibrin (Najeeb et al., 2017; Ghanaati et al., 2019), and concentrated growth factors (Wang et al., 2021b), have promising effects that improve soft-tissue healing. A previous clinical trial evaluated the effect of platelet-rich plasma on Ti mesh exposure. In the study, 15 patients were recruited and underwent alveolar bone augmentation with Ti mesh and platelet-rich plasma, and another 15 patients underwent alveolar bone augmentation only with Ti mesh. After a 6 month follow-up, the patients were recalled for evaluation and subsequent treatment. No exposure was observed in the platelet-rich plasma group compared with the group without platelet-rich plasma (Torres et al., 2010). This study showed that autologous bioactive materials contribute to soft-tissue healing. For wound dehiscence prevention, Masayuki Takano et al. reported a minimally invasive subperiosteal tunneling flap technique involving long labial incision, cervical palatal incision, and peritoneal-releasing incision (Takano et al., 2019). Although some studies showed that the premature (within 4–6 weeks) or delayed (after 6 weeks) exposure of Ti mesh has no effect on bone augmentation in the presence of a pseudo-periosteum (Ciocca et al., 2018), infection does compromise bone augmentation, and timely measures are still needed when exposure occurs, such as gentle cleaning and daily rinsing with 0.12% chlorhexidine or removing prematurely exposed meshes (Hartmann et al., 2019; Belleggia, 2021).

These results indicate that a customized mesh is round and blunt and more suitable for alveolar bone morphology, and they show low exposure rates. Selecting an appropriate surgical procedure and combining autologous bioactive materials can promote wound healing and reduce Ti mesh exposure. When a Ti mesh is exposed, timely measures are needed to control infection and prevent bone graft failure.

#### 4.1.3 Advantages and Disadvantages of a Customized Ti Mesh

Apart from stabilizing graft material, creating enough space, and acting as a barrier membrane to guide bone regeneration in the same manner as a traditional Ti mesh does, patient-specific Ti, PEEK, and uHA/PLLA meshes have several benefits, as shown in Table 2. Besides, a customized Ti mesh is thinner and has more extensive clinical applications compared with PEEK and uHA/PLLA.

**TABLE 2:** Comparison of three customized barrier membranes in GBR.

Category of customized membranes	Common advantages	Common disadvantages	Specific advantages	Disadvantages
Titanium mesh	1) Suitable for various bone defects, especially complex large bone defects	Customized barrier membranes alone cannot prevent soft tissue ingrowth due to the pores, and the formation of pseudo-periosteum occupies osteogenic space and weakens osteogenic effect. (The formation of pseudo-periosteum beneath the PEEK and uHA/PLLA-based customized barrier has not been reported due to the lack of relevant literature.)	Thin and extensively applied in clinical usage	Radiopacity, high exposure rate and the need for secondary removal
	2) Easy to determine bone volume for bone reconstruction and facilitate bone-grafting design			
PEEK mesh	3) More suitable for jaw anatomical morphology		Radiolucent and has good tensile strength and elasticity similar to human bone with less mucous membrane irritation	Thick, costly, non-osteoconductive and needs a secondary removal. (Whether PEEK can reduce mesh exposure rate remains to be studied.)
	4) Avoid manual shaping during the operation, which greatly shorten the during time			
uHA/PLLA mesh	5) The smooth external shape is conductive to fixation and secondary removal, which can reduce mucosal irritation and exposure time		Osteoconductive, radiolucent, bioresorbable and doesn’t require a second surgical removal and has an elastic modulus similar to human bone	Thick and needs complicated production process. (Relevant literature is insufficient and further research is still needed.)
	6) Reduce the burden on surgeons and differences between different surgeons			

Customized Ti, PEEK, and uHA/PLLA barrier membranes are designed with pores to receive nutrients and blood from the periosteum to promote bone healing. However, soft tissue-derived cells can grow through the pores into the osteogenic region and weaken the osteogenic effect. Therefore, using a customized barrier membrane alone cannot prevent soft tissue ingrowth and pseudo-periosteum formation. More research is needed to address this limitation.

Customized Ti mesh also has its own disadvantages to address. Compared with traditional Ti mesh, customized Ti mesh can cause less membrane exposure because of its smooth round blunt shape. However, in some cases, the exposure rate of customized Ti mesh may reach an average of 20% or higher due to inherent stiffness (Sagheb et al., 2017). Besides, Ti mesh has no antibacterial ability and may need to be removed in the early stage of membrane exposure to avoid bone graft failure (Hartmann et al., 2019). A secondary surgery is still required to remove the Ti mesh (Zhou et al., 2021). In addition, the radiopacity of a Ti mesh may affect the imaging results of postoperative X-ray examinations.

To sum up, although customized Ti meshes are widely applied in GBR for alveolar bone augmentation and maxillofacial bone reconstruction, their inherent traits can lead to clinical complications and increase the complexity of treatment procedures. Relevant research should be directed towards increasing the antibacterial ability and osteogenic ability of Ti mesh to reduce membrane exposure and pseudo-periosteum formation.

### 4.2 PEEK-Based Customized Barrier Membrane

#### 4.2.1 Mechanical and Biological Properties of PEEK

PEEK is a highly compatible polyaromatic semi-crystalline thermoplastic polymer (Gu et al., 2020; Ma et al., 2020) and has been approved by the US FDA Drug & Device Master File. *In vitro* experiments found no evidence of the mutagenic or cytotoxic effects of PEEK (Katzer et al., 2002). It has excellent mechanical properties, with an elastic modulus of 3.6 GPa, which can be increased to 18 GPa with the addition of carbon fiber, which is similar to the elastic modulus of cortical bone at 15 GPa. Furthermore, it has sufficient radiolucency and hardness, showing a flexural strength of 140–170 MPa to resist masticatory pressure (Schwitalla and Müller, 2013). These results reveal that PEEK has considerable biocompatibility and mechanical properties and can be used in repairing bone defects.

#### 4.2.2 Fabrication, Clinical Application, and Limitations of PEEK

Patient-specific PEEK devices are virtually designed with specific software and fabricated from PEEK blocks with milling machines (El Morsy et al., 2020). Apart from cutting a scaffold, a fused deposition modeling 3D printer can also deposit customized PEEK scaffolds layer by layer with molten PEEK (Li et al., 2022).The devices are sterilized through immersion in 2.4% glutaraldehyde prior to surgery.

In the craniofacial field, PEEK has been widely used as a customized implant for repairing large skull defects (van de Vijfeijken et al., 2019; Yang et al., 2020) and mandibular reconstruction (Atef et al., 2021; Kang et al., 2021), and is often compared with Ti meshes in terms of price and operative time (Binhammer et al., 2020). Although customized PEEK implants can reduce operative duration to a certain extent compared with manually bent Ti meshes, they are more costly and are associated with an increased number of complications, such as infection (Rosinski et al., 2020).

**FIGURE 6:**
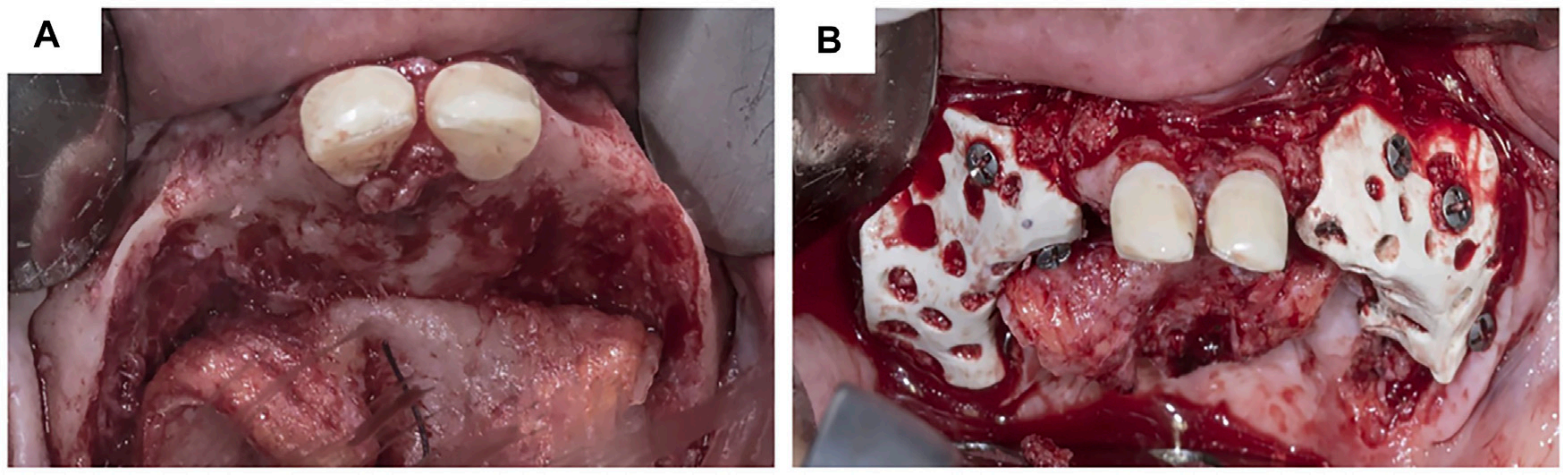
Customized PEEK mesh in clinical usage. **(A)** Customized PEEK mesh. **(B)** Fixation of customized PEEK mesh. Reproduced with permission from El Morsy et al. (2020).

As shown in Table 1 and Figure 6, PEEK has been successfully used in augmenting atrophied alveolar ridges with vertical and horizontal bone gains of 3.47 mm (±1.46) and 3.42 (±1.1), respectively (El Morsy et al., 2020). In the formation of new bone mass, no statistical difference was found between prebent Ti mesh and patient-specific PEEK (Mounir et al., 2019). These results suggest that customized PEEK membranes can successfully augment severely atrophied alveolar bones. However, clinical samples are few, and given that PEEK does not possess any features of osteogenesis or osseointegration (Zhao et al., 2021), it cannot completely replace Ti meshes.

#### 4.2.3 Advantages and Disadvantages of a Customized PEEK Mesh

Customized PEEK membranes have the same benefits as customized Ti meshes (Table 2). The above results indicate that PEEK has better tensile strength and elasticity than Ti, is more similar to human bone, and results in less mucous membrane irritation, which may reduce membrane exposure to some extent (Mounir et al., 2019). The radiographic property of a PEEK membrane has no effect on X-ray imaging. However, as PEEK is non-absorbable and non-osteoconductive and needs to be removed in a second operation (Zhao et al., 2021), it may not completely replace Ti mesh, and its high clinical cost may limit its potential use (Rosinski et al., 2020). Further studies should focus on enhancing its osteogenic and osseointegration ability as well as production efficiency problems.

### 4.3 uHA/PLLA-Based Customized Barrier Membrane

#### 4.3.1 Properties and Advantages of uHA/PLLA

uHA/PLLA is a third-generation bio-resorbable material in oral and maxillo-facial medicine. It is processed with a specific forging protocol that combines uncalcined and u-HA particles (30 and 40% by weight) with PLLA (Shikinami and Okuno, 1999). PLLA belongs to the first generation of bioresorbable materials in osteosynthesis surgery. However, several disadvantages limit its potential use in maxillofacial medicine. First, insufficient strength and lack of osteoconduction properties make it unsuitable for stress-bearing zones. In addition, acidic degradation products can elicit an inflammatory reaction. Its crystallinity and hydrophobicity prevent its hydrolysis during the first 2 years, increasing resorption duration and delaying tissue degradation reactions (Kanno et al., 2018). Hydroxyapatite is radiopaque and osteoconductive and has been used as bone grafts in mandibular surgery. However, its high absorption rate, high infection rate, and inherent brittleness have limited its clinical application. uHA-PLLA combines the strengths of both materials through a unique forging process and offsets their respective weaknesses. uHA confers osteoconductivity on PLLA and enhances its strength (Shikinami and Okuno, 1999). uHA-PLLA has a mild hydrolysis reaction rate and can stably release PLLA fragments without causing tissue swelling, and thus it has been widely used in orthodontic treatment (Shikinami et al., 2005).

uHA-PLLA composites possess excellent biocompatibility and osteoconductivity and have been used in bone-fixation systems, such as plates and screws (Ueki et al., 2011). Yasuo Shikinami et al. compared F-PLLA-only rods with F-u-HA 30/40 rods implanted in a rabbit bone cavity and found that the F-PLLA-only group elicited an inflammatory response in the body because of the uneven release of PLLA particles. The F-u-HA 30/40 rods did not induce any adverse effects *in vivo*. These results showed that the uHA-PLLA composite is highly biocompatible (Shikinami et al., 2005). The uHA particles conferred osteoconductivity on PLLA, and newly formed bone was observed in uHA-PLLA sheets covering critical size defects compared with the PLLA in the rat model (Dong et al., 2019). A previous study confirmed that uHA-PLLA meshes are as effective as Ti meshes in bone augmentation (Moroi et al., 2013).

In addition, uHA-PLLA has excellent mechanical traits, with a modulus of 12 GPa, which is close to that of a cortical bone. The bending strength is 270 MPa, which is higher than that of PLLA and even higher than that of a human cortical bone. After 24 weeks, it can be maintained at 200 MPa, which is sufficient for normal bone regeneration (Shikinami and Okuno, 1999). Moreover, degradation *in vivo* takes 3–5 years, which is sufficient for bone healing, particularly the correction of large mandibular defects (Shikinami et al., 2005). These results show that uHA/PLLA combines the strengths of PLLA and hydroxyapatite, possessing excellent mechanical traits, biocompatibility, and osteoconductivity, and can be applied to bone tissue reconstruction.

#### 4.3.2 Fabrication, Clinical Application and Prospect of uHA/PLLA Based Customized Barrier Membrane

Customized uHA/PLLA barrier membranes are usually prefabricated with the same fabrication method used for customized Ti meshes, that is, manipulating and bending commercial uHA/PLLA plates on stereolithographic models. A uHA/PLLA plate becomes soft after immersion in hot water at 60°C and is easily shaped with tweezers, but hardens when the temperature returns to 25°C (Moroi et al., 2013).

**FIGURE 7:**
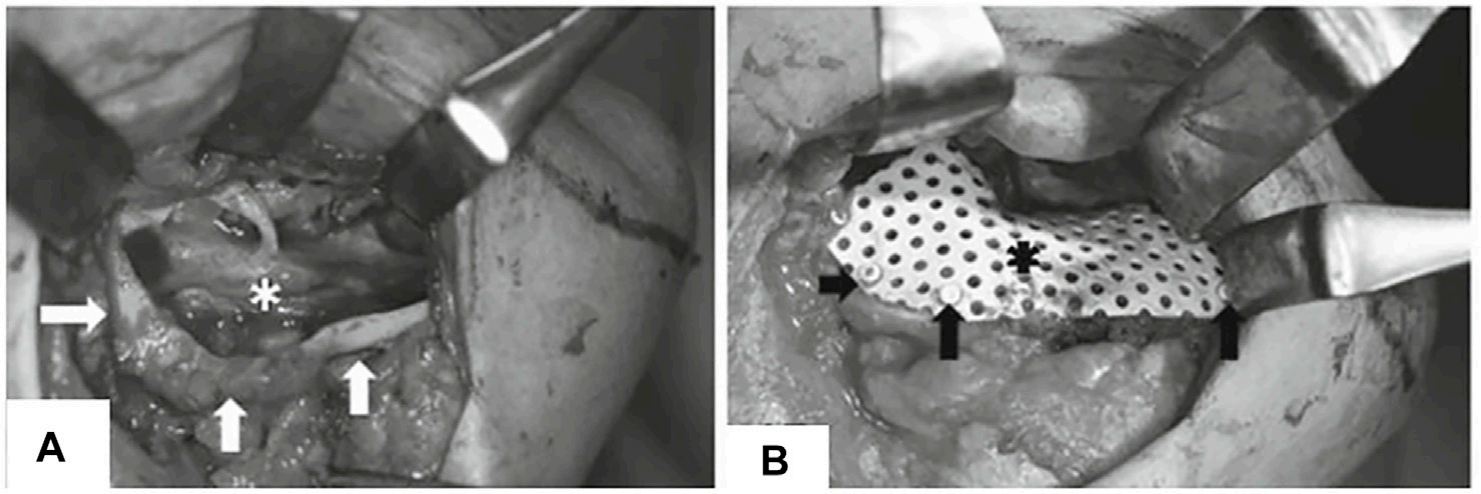
Customized uHA-PLLA mesh in clinical usage. **(A)** Exposure of alveolar bone ridge. **(B)** Fixation of customized uHA-PLLA mesh. Reproduced with permission from Matsuo et al. (2010).

Akira Matsuo et al. fabricated a custom uHA-PLLA mesh tray for mandibular reconstruction by prototyping a mandibular stereolithography model and preforming HA-PLLA sheets based on that model to conform to a 3D contour (Figure 7) (Matsuo et al., 2010). One patient in their case report received dental implants 10 months after surgery and was followed up for 1 year. They obtained reliable and stable results. Although the duration from bone grafting to implant placement was insufficient to fully degrade uHA-PLLA, the alveolar part was easily removed, and the external part of the tray was preserved and suitable for implant placement. However, owing to the limited number of cases and short follow-up time, the result was not convincing (Table 1) (Matsuo et al., 2010). Akira Matsuo et al. assessed the fitness of a manually bent 0.8 mm-thick uHA-PLLA trays in dog models and revealed that the uHA-PLLA tray had a better fit to the lingual side of the alveolar bone than the manually bent Ti mesh, and no statistical difference was found on the buccal side. The CT value of the newly formed bone under the uHA-PLLA mesh was higher than that of the Ti mesh and was close to that of residual bone (Matsuo et al., 2012). These results show that a customized uHA-PLLA mesh can closely match an alveolar bone, maintain stable space, and achieve predictable bone regeneration. However, a few studies have reported the application of uHA-PLLA in GBR and 3D printing. More research is needed to explore its potential use in maxillofacial reconstruction and alveolar bone augmentation.

#### 4.3.3 Advantages and Disadvantages of Customized uHA-PLLA Mesh

Bioresorbable materials with stiff strength and radiolucency, such as uHA-PLLA, may provide an alternative for predictable bone augmentation and the fabrication of customized barrier membranes (Table 2). A bioresorbable trait does not allow it to be removed in a second surgery. However, processes for manipulating and bending commercial uHA/PLLA plates on stereolithographic models (Moroi et al., 2013) are more complicated than processes for directly prototyping them, and relevant literature is insufficient. In summary, customized Ti devices are still the most mainstream protocol in GBR.

## 5. Current Progress and Future Directions

### 5.1 Potential Solutions to Reduce Soft Tissue Ingrowth

Soft tissue ingrowth occupies osteogenic space and subsequently impairs the osteogenic effect. A non-porous barrier membrane can avoid fibrous tissue formation, but insufficient blood supply may delay bone regeneration (Guo et al., 2020). More research is needed on how to avoid soft tissue ingrowth for customized barrier membranes with pores. To date, numerous studies are trying to overcome this limitation. Collagen membranes have good soft tissue reactions and can reduce the migration of epithelial cells into the bone defects. Combined use of customized membranes and collagen membranes during GBR could ensure greater predictability with reduced soft tissue interposition (Borges et al., 2020). Surface modification could also help to reduce the growth of connective tissue. Nguyen et al. demonstrated that heat-treated Ti mesh subjected to anodization and cyclic precalcification could attach directly to the bone. However, connective tissues were found to grow between untreated Ti mesh and bone (Nguyen et al., 2016). Besides, cell occlusion effects are also affected by differences in superficial topography. It was found that the rough surface of an absorbable barrier membrane had more giant cells attached to it than the smooth surface contained more inflammatory cells, indicating that different surface topographies promote differential soft tissue responses (de Santana et al., 2010). Furthermore, Gutta and others demonstrated that macroporous Ti mesh (pore size 1.2 mm) significantly hindered soft tissue growth compared to microporous mesh (pore size 0.6 mm) (Gutta et al., 2009), suggesting that soft tissue ingrowth could be suppressed by changing the pore size. In addition, inspired by “Janus”, a Janus membrane with the upper layer blocking the migration of fibroblasts and the bottom side promoting osteogenesis also provides researchers with a new direction (He et al., 2022).

Based on existing studies, several potential solutions can be suggested to reduce soft tissue ingrowth: 1) combined use of customized membrane with collagen membrane; 2) using screws to fix the customized membrane to prevent micromovement between the bone graft and the membrane; 3) pore size and surface topography optimization; 4) surface modification of membrane materials; 5) development and application of Janus membranes. Besides, in order to obtain sufficient bone mass, excessive bone augmentation can avoid insufficient bone volume caused by soft tissue ingrowth and pseudo-periosteum formation.

### 5.2 Enhancement of Bioactive Properties

Along with the progress of biomaterials, research for improving the bioactive properties of Ti alloys is advancing (Liu et al., 2020; Wang et al., 2021a; Wei et al., 2021). Xu et al. focused on the study of Cu ions with excellent antibacterial properties. By incorporating Cu ions into Ti alloys, they found that Ti6AL4V alloy meshes offer broad prospects for clinical application and exert considerable pro-angiogenic and anti-inflammatory effects (Xu et al., 2018). Thuy-Duong Thi Nguyen et al. coated Ti meshes with strontium-substituted calcium phosphate for surface modification and achieved better osseointegration compared with that in the untreated group in rat calvarial defect models (Nguyen et al., 2019). Zhao et al. found that porous chitosan gelatin doxycycline coatings on the Ti mesh exhibited antibacterial effects (Zhao et al., 2022). The improvement of bioactive properties can enhance the bone-binding and antibacterial abilities of Ti mesh, which may help reduce the risk of exposure.

The absence of osteogenesis and osseointegration ability limits the clinical application of PEEK (Han et al., 2022), so numerous strategies have been proposed to strengthen its bioactivity. In recent years, PEEK implants have gone through three development phases: 1) mechanical property enhancement; 2) cytocompatibility and osteogenic ability enhancement; and 3) osseointegration and anti-inflammatory enhancement (Gu et al., 2020). Improvement in PEEK properties is usually achieved through surface modification (Frankenberger et al., 2021; Mehdizadeh Omrani et al., 2021; Jiang et al., 2022) and blending modification (Ma et al., 2020; Ren et al., 2020; Hu et al., 2022). Waser-Althaus et al. used oxygen and ammonia plasma to modify PEEK surfaces and enhance their hydrophilicity and protein adsorption capacity. *In vitro* tests showed that compared with pure PEEK, modified PEEK showed stronger osteogenic ability and could better promote the osteogenic differentiation of adipose-derived mesenchymal stem cells (Waser-Althaus et al., 2014). It was found that adding silicon nitride powder within a PEEK matrix can significantly enhance its osteoconductive and bacteriostatic properties (Pezzotti et al., 2018). Overall, endowing PEEK with antibacterial and bone-binding capabilities and applying it to GBR will certainly promote wound healing, reduce the barrier membrane exposure rate, and improve osteogenesis. In view of numerous articles about PEEK modification, a series of factors, such as complexity of processing, economic cost, and clinical practicability, must be considered in its future development.

### 5.3 Development of Biodegradable Materials

Biomaterials for tissue engineering are evolving from the first generation of metals and ceramics to the second generation of bioceramics (Brunello et al., 2020) and polymers (Babilotte et al., 2019) and to the third generation of absorbable metal materials (Rahman et al., 2020). In recent years, studies about biodegradable metal materials with excellent mechanical properties, such as magnesium-based alloys (Chen et al., 2018; Rahman et al., 2020; Yan et al., 2022) and zinc-based alloys (Jia et al., 2020), have grown dramatically. Magnesium-based alloys have good biocompatibility and mechanical properties and offset the rapid degradation of magnesium and hydrogen production. Asgari et al. prepared a magnesium oxide film coating on a Mg-based substrate and established a rat calvarial defect model. Their results revealed the improved osteo-compatibility and biodegradability of magnesium alloys (Asgari et al., 2019). Zinc-based alloys can improve the low strength and brittleness of pure zinc and provide a novel biodegradable material for orthopedic applications. For example, zinc-manganese biodegradable metals have been utilized in animal bone defect models and successfully promoted bone regeneration (Jia et al., 2020). These results suggest that the development of biodegradable materials can well solve the problem of secondary removal of non-absorbable membranes after GBR. Barrier membranes made of biodegradable materials should have good biocompatibility, good mechanical properties to maintain space, excellent properties to promote osteogenesis and reduce the risk of exposure. These properties will provide a promising prospect for GBR (Toledano-Osorio et al., 2021).

## 6 Conclusion

Developments in digital modeling and 3D printing technologies have produced materials, such as Ti alloy, PEEK, and uHA/PLLA, with excellent mechanical properties and good biocompatibility, which are used in fabricating customized bone regeneration sheets for GBR. These materials are predictable and stable solutions for maxillofacial reconstruction and alveolar bone augmentation. By comparison, little research has been performed on customized PEEK and uHA-PLLA meshes, and a customized Ti mesh is the most mainstream protocol in GBR, although it needs to be removed in a second operation. In addition, improvement of material properties endows Ti and PEEK with high antibacterial and osteogenic properties, and the emergence of novel biological materials is developing towards biological absorption, enhanced osteogenesis, and reduced membrane exposure, which will promote the application of customized absorbable barrier membranes in GBR.
